# Association of Urinary Levels of Bisphenols A, F, and S with Endometriosis Risk: Preliminary Results of the EndEA Study

**DOI:** 10.3390/ijerph17041194

**Published:** 2020-02-13

**Authors:** Francisco M. Peinado, Inmaculada Lendínez, Rafael Sotelo, Luz M. Iribarne-Durán, Jorge Fernández-Parra, Fernando Vela-Soria, Nicolás Olea, Mariana F. Fernández, Carmen Freire, Josefa León, Beatriz Pérez-Cabrera, Olga Ocón-Hernández, Francisco Artacho-Cordón

**Affiliations:** 1 Instituto de Investigación Biosanitaria ibs, E-18012 Granada, Spain; franciscopeinado@correo.ugr.es (F.M.P.); luzmaria.irdu@gmail.com (L.M.I.-D.); fervs@ugr.es (F.V.-S.); nolea@ugr.es (N.O.); marieta@ugr.es (M.F.F.); cfreire@ugr.es (C.F.); pepileon@ugr.es (J.L.); 2 General Surgery, San Cecilio University Hospital, E-18016 Granada, Spain; inmalendinez10@gmail.com (I.L.); bperezcabrera@ugr.es (B.P.-C.); 3 Gynaecology and Obstetrics Unit, ‘San Cecilio’ University Hospital, E-18016 Granada, Spain; hre459@gmail.com; 4 Gynaecology and Obstetrics Unit, ‘Virgen de las Nieves’ University Hospital, E-18014 Granada, Spain; jfdzparra@gmail.com; 5 CIBER de Epidemiología y Salud Pública (CIBERESP), E-28029 Madrid, Spain; 6 Radiology and Physical Medicine Department, University of Granada, E-18016 Granada, Spain; 7 Nuclear Medicine Unit, ‘San Cecilio’ University Hospital, E-18016 Granada, Spain; 8 Digestive Medicine Unit, ‘San Cecilio’ University Hospital, E-18012 Granada, Spain; 9 CIBER Enfermedades Hepáticas y Digestivas (CIBEREHD), E-28029 Madrid, Spain

**Keywords:** bisphenol A, bisphenol F, bisphenol S, endocrine disruption, endometriosis, oxidative stress

## Abstract

*Aim:* The aim of this study was to explore associations of urinary concentrations of bisphenols A (BPA), S (BPS), and F (BPF) and of thiobarbituric acid reactive substances (TBARS) with the risk of endometriosis in women of childbearing age. *Methods*: This case–control study enrolled 124 women between January 2018 and July 2019: 35 women with endometriosis (cases) and 89 women without endometriosis undergoing abdominal surgery for other reasons (controls). Endometriosis was diagnosed (cases) or ruled out (controls) by laparoscopic inspection of the pelvis and the biopsy of suspected lesions (histological diagnosis). Fasting urine samples were collected before surgery to determine concentrations of BPA, BPS, BPF, and TBARS. Associations of bisphenol and TBARS concentrations with endometriosis risk were explored with multivariate logistic and linear regression analyses. *Results:* After adjustment for urinary creatinine, age, BMI, parity, and residence, endometriosis risk was increased with each 1 log unit of BPA [OR 1.5; 95%CI 1.0–2.3] and Σbisphenols [OR 1.5; 95%CI 0.9–2.3] but was not associated with the presence of BPS and BPF. Classification of the women by tertiles of exposure revealed statistically significant associations between endometriosis risk and the second tertile of exposure to BPA [OR 3.7; 95%CI 1.3–10.3] and Σbisphenols [OR 5.4; 95%CI 1.9–15.6]. In addition, TBARS concentrations showed a close-to-significant relationship with increased endometriosis risk [OR 1.6; 95%CI 1.0–2.8], and classification by TBARS concentration tertile revealed that the association between endometriosis risk and concentrations of BPA [OR 2.0; 95%CI 1.0–4.1] and Σbisphenols [OR 2.2; 95%CI 1.0–4.6] was only statistically significant for women in the highest TBARS tertile (>4.23 μM). *Conclusion*: Exposure to bisphenols may increase the risk of endometriosis, and oxidative stress may play a crucial role in this association. Further studies are warranted to verify these findings.

## 1. Introduction

Endometriosis is a gynecological disorder characterized by the growth of endometrial-like tissue outside the uterine cavity, e.g., on fallopian tubes, ovaries, peritoneal wall, and/or intestine [1]. This ectopic endometrium is sensitive to hormonal signaling, usually undergoing histological changes along the menstrual cycle, which leads to such symptoms as dysmenorrhea, dyspareunia, chronic pelvic pain, and menstrual irregularities [2]. Further complications can include reduced fertility, infertility, problems during pregnancy and delivery, increased risk of coronary heart disease, and higher long-term risk of gynecological cancer [3,4]. Endometriosis is considered to be one of the most frequent pathologic conditions in women, despite the absence of national registries and the numerous women with this disease who are free of symptoms. A recent study carried out in Israel reported an incidence of endometriosis of 7.2 per 10.000 women aged 15–55 years, with an annual increase in its incidence of 1.6% [5]. However, there is a scarcity of information regarding prevalence or incidence of endometriosis in Spain.

Little is known about the etiology of endometriosis, although its relationship with retrograde menstruation, coelomic metaplasia, and/or lymphatic and vascular metastasis has been hypothesized [6]. However, none of these hypotheses fully explain the development of these ectopic endometrial lesions [6], and there remains a need to identify potential risk factors for this disease. Besides genetic susceptibility [7,8], environmental factors have been proposed as crucial orchestrators of endometriosis development [9]. For instance, exposure to so-called endocrine-disrupting chemicals (EDCs) may be a potential risk factor, given the estrogen-sensitivity of endometrial tissue and the daily widespread exposure of humans to these compounds [9]. Known EDCs include bisphenol A (BPA), a high-production-volume chemical used in the manufacture of polycarbonates and epoxy resins for a wide range of plastic products, such as water bottles, plastic containers, and cans for food or beverages [10]. The global volume of BPA consumption was estimated at 7.7 million metric tons in 2015 and is expected to reach 10.6 million metric tons in 2022 [11]. The leaching of BPA from these materials into food is considered mainly responsible for human exposure to BPA [12], although there have been recent reports on exposure from other sources, including personal care products [13], clothes [14,15,16], and thermal paper [17]. It is suspected that human exposure to BPA may be associated with the development of estrogen-dependent diseases, given its xenoestrogenic properties [18,19]. Rising public concerns over the past decade has led to the utilization of bisphenol S (BPS) or bisphenol F (BPF) instead of bisphenol A in the manufacture of plastic products [20], although these alternatives have been found to have similar xenoestrogenic potential in in vitro studies [21,22]. In addition to hormonal disruption, it has been observed that not only BPA but also these substitutes can induce a misbalance in redox status [23,24,25], which has been described as a crucial risk factor for endometriosis [26]. In this regard, oxidative stress has been implicated in the development [27], progression [27,28], and severity [29] of endometriosis through the disruption of related signaling pathways.

Specifically, BPA exposure has been associated with polycystic ovary syndrome [30], ovarian insufficiency [31], and infertility [32]. In regard to endometriosis, an in vivo study reported changes in endometrial lesions after exposure to BPA or its analogues [33], although a recent meta-analysis revealed only limited and contradictory epidemiological evidence on the association between BPA exposure and endometriosis risk [34]. By contrast, there has been no published research on the association between exposure to BPS or BPF and endometriosis. The objective of this study was to explore associations of urinary concentrations of BPA, BPS, BPF, and thiobarbituric acid reactive substances (TBARS) with the risk of endometriosis in women of childbearing age. Finally, we assessed the influence of oxidative lipid damage on the association between bisphenol exposure and endometriosis. 

## 2. Material and Methods

### 2.1. Study Population and Sample Collection

This preliminary study is part of the EndEA Study (Endometriosis y Exposición Ambiental), a hospital-based case–control study designed to assess environmental factors related to endometriosis and its potential mechanisms of action. From January 2018 to July 2019, 124 women—35 with endometriosis (cases) and 89 controls—were enrolled in the present study from among patients of the Surgery and the Gynecology and Obstetrics Units of the San Cecilio and Virgen de las Nieves University Hospitals in Granada, southern Spain. Cases were women with endometriosis diagnosed by laparotomy or laparoscopic surgery and histological confirmation, while controls were women undergoing abdominal surgery for non-malign diseases (including acute appendicitis, biliary disease, hiatus hernia, ovarian torsion, corpus luteum, uterine fibroids, and cystadenomas, among others), in whom the absence of endometrial lesions was visually and histologically confirmed. Inclusion criteria for both cases and controls were: premenopausal woman aged between 20 and 54 years, receipt of abdominal surgery, and body mass index (BMI) below 35 kg/m^2^. Exclusion criteria were: history of cancer (except non-melanoma skin cancer), pregnancy at study enrolment, and inability to read and sign the informed consent document. Cases were paired with controls by frequency. Patients were invited to participate in the study by clinicians involved in the research, who explained its principles and aims. Written informed consent was provided by all participants in the study. The study was approved by the Research Ethics Committee of Granada (0464-N−18). All study data were coded for confidentiality.

In accordance with the EHPect project in which our research group participates (http://endometriosisfoundation.org/ephect/), standard operating procedures were used for the banking of biological samples and the standardized questionnaires for data gathering. Spot first-morning urine samples were collected in 10 mL of BPA-free glass tubes before surgery and immediately stored at −80 °C until further analysis at the Biobank of the Public Andalusian Healthcare System. All participants underwent clinical and anthropometrical examination, calculating their BMI from their height and weight. EHPect surgical and clinical questionnaires were used to gather sociodemographic, lifestyle, clinical, and surgical data, including residence (urban or suburban), educational level (university degree or less), occupational status (working outside of the home or not), current smoking (yes or no), parity (nulliparous, primiparous, or multiparous), and average level of menstrual bleeding (mild, moderate, or severe). Clinicians also completed standardized operative reports on primary and secondary diagnoses, and endometriosis was staged according to the Revised American Fertility Society classification [35].

### 2.2. Sample Extraction and Chemical Analysis

Dispersive liquid–liquid microextraction (DLLME) and ultra-high performance liquid chromatography with tandem mass spectrometry detection (UHPLC-MS/MS) were used to determine urinary concentrations of BPA, BPF, and BPS, as previously described [36]. All reagents were analytical grade unless otherwise specified. Urine samples were thawed at room temperature and centrifuged at 2600× *g* for 10 min, taking 1.0 mL for analysis. An enzymatic solution of β-glucuronidase/sulfatase was prepared daily, dissolving 10 mg of β-glucuronidase/sulfatase (3 106 U g solid 1) in 15 mL of 1M ammonium acetate/acetic acid buffer solution (pH 5.0). Each sample was enriched with 50 µL of enzyme solution to determine the total amounts (free and conjugated) of BPA, BPF, and BPS in urine and incubated for 24 h at 37 °C. Samples were then placed in 15 mL glass tubes, followed by the addition of 20 µL of standard replacement solution (5 mg/L of EP−^13^C_6_, 2 mg/L of BPA-D_16_, and 2 mg/L of BP-d_10_) and dilution with 10 mL of 10% aqueous NaCl solution (pH 2.0, adjusted with 0.5 M HCl). Samples were then mixed with 0.5 mL acetone (dispersing solvent) and 1.0 mL trichloromethane (extraction solvent), manually shaken for 30 s, and centrifuged for 10 min at 2600× *g*. The entire volume of the sedimented phase was transferred to a glass vial using a 1.0 mL pipette, and the organic phase was evaporated under a nitrogen stream. The residue was dissolved with 100 µL of an acetonitrile/water mixture (0.1% ammonia, 70:30 (*v*/*v*)) and vortexed for 30 s. The extract was then ready for analysis by UHPLC-MS/MS. The limit of detection (LOD) was determined as the minimum detectable amount of analyte with a signal-to-noise ratio (SNR) ≥3. The LODs obtained were 0.12 ng/mL for BPA and BPF, and 0.20 mg/mL for BPS.

### 2.3. Oxidative Stress Biomarkers

Lipid peroxidation was assessed by the quantification of TBARS in urine using the Colorimetric TBARS Microplate Assay Kit, a commercial ELISA enzymatic kit supplied by Oxford Biomedical Research (Rochester Hills, MI, USA). This method is based on the reaction of 2-thiobarbituric acid with MDA (compound resulting from the decomposition of polyunsaturated fatty acid lipid peroxides) at 25 °C to yield a chromophore with maximum absorbance at 532 nm. The LOD of TBARS was 1.0 μM.

### 2.4. Statistical Analysis

The sociodemographic, lifestyle, and gynecological characteristics of cases and controls were expressed as means with standard deviation or as percentages, depending on the continuous or categorical nature of the variable. Urinary concentrations of bisphenols (BPA, BPS, BPF, and the sum of bisphenols (∑bisphenols)) and TBARS were expressed as geometric means (GMs) with geometric standard deviation (GSD), as minimum and maximum values, and as percentiles (25, 50, and 75). Urinary concentrations of bisphenols and TBARS below the LOD were assigned a value of LOD/2. The Shapiro-Wilk test was used to explore the normality of continuous variables, and logarithmic transformation was carried out in those that did not fulfil normality criteria.

Differences in characteristics between cases and controls were explored using the chi-square test for categorical variables and the parametric Student’s *t*-test/ANOVA or non-parametric Mann–Whitney/Kruskal–Wallis tests for continuous variables. Differences in urinary concentrations of bisphenols between cases and controls were assessed through the Mann–Whitney test, while differences between controls, cases staged I/II, and cases staged III/IV were explored with Kruskal–Wallis and Jonckheere–Terpstra tests, while trends across Spearman’s test were used to evaluate linear correlations between bisphenol and TBARS concentrations in urine. The relationship between urinary bisphenol levels and endometriosis risk was assessed by unconditional logistic regression analyses, entering BPA and Σbisphenol concentrations as log-transformed continuous variables and entering BPS and BPF concentrations with a detection frequency below 30% as categorical variables (>LOD vs. <LOD). Associations with BPA and Σbisphenols were further examined by entering bisphenol concentrations classified in tertiles. Given the limited sample size, regression analyses were sequentially adjusted for (1) urinary creatinine; (2) urinary creatinine, age, and BMI; and (3) urinary creatinine, age, BMI, residence, and parity. The selection of covariates was based on previous reports [37,38,39]. Odds ratios (ORs) with 95% confidence intervals (95% CIs) were calculated for the risk of endometriosis, considering bisphenol concentrations to be significantly associated with this risk when 95% CIs of the OR in the adjusted models did not overlap the null value (1). Estimates reflect the odds of endometriosis risk for each 1 log unit of bisphenol or TBARS concentrations. A similar approach was used to explore the association between urinary TBARS concentrations and the risk of endometriosis. To test the trend across tertiles of bisphenol/TBARS concentrations, continuous variables were created by assigning values equal to the median tertile concentration in each category. This variable was entered in the adjusted logistic regression model, and the accompanying *p*-value was interpreted.

The influence of lipid oxidative status on the association between concentrations of bisphenols and endometriosis was first studied by analyzing the association between bisphenol concentrations and TBARS, using linear regression models adjusted for urinary creatinine, age, BMI, residence, and parity and presenting regression estimates as exp(β) with corresponding 95% CIs. Next, we conducted adjusted unconditional logistic regression analyses of the association between bisphenol concentrations and endometriosis by tertiles of TBARS values.

Data were stored and processed using SPSS Statistics 23.0 (IBM, Chicago, IL, USA) and R statistical computing environment v3.1 (http://www.r-project.org/). The calculated power (1-β) of the statistical analysis to detect an OR ≥2 assuming an α-error of 0.05 was 0.54.

## 3. Results

Sociodemographic, lifestyle, and clinical characteristics of cases and controls are summarized in Table 1. No statistically significant differences were observed between cases and controls in terms of age, BMI, residence, educational level, occupational status, smoking habits, parity, or level of menstruation bleeding. Among the 35 cases, 23 (65.7%) were diagnosed with endometriosis stage I/II, while endometriomas were deeply infiltrated in 11 (31.4%).

Urinary concentrations of BPA, BPS, BPF, and Σbisphenols are displayed in Table 2. BPA was the most frequently detected bisphenol compound in cases (100.0%) and controls (88.5%), followed by BPF (in 28.6% of cases and 29.9% of controls), and BPS (in 11.4% of cases and 16.1% of controls). The GM concentration of BPA was 5.5 ng/mL (25–75th percentile: 4.0–8.4 ng/mL) in cases and 3.0 ng/mL (25–75th percentile: 1.1–8.8 ng/mL) in controls, the GM BPF concentration was <LOD (<LOD−0.2 ng/mL) in both cases and controls, and the GM BPS concentration was <LOD (<LOD-<LOD) in both cases and controls. The GM concentration of Σbisphenols was higher in cases (5.9 ng/mL) than in controls (3.8 ng/mL). However, these differences in bisphenol concentrations were not statistically significant. No differences were observed in cases according to the stage (III/IV vs. I/II) (Supplementary Table S1).

**Figure 1:**
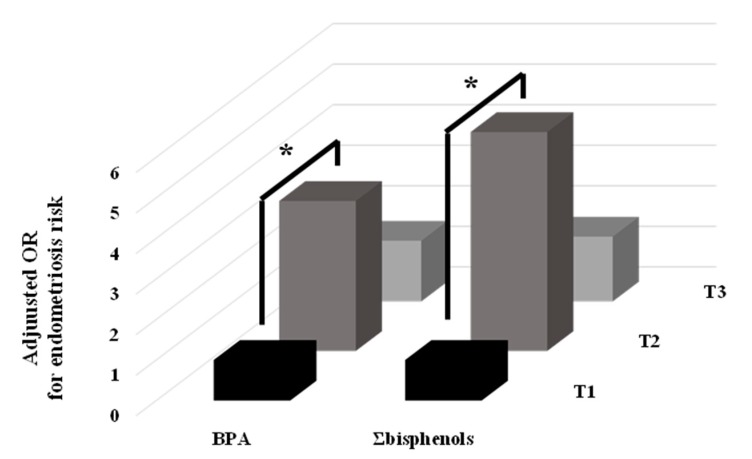
Adjusted odds ratios (OR) for endometriosis in the tertiles (T) of urinary concentration of BPA and Σbisphenols. * *p*-value < 0.050.

After adjusting for urinary creatinine, age, BMI, parity, and residence, urinary BPA concentrations were significantly associated with an increased risk of endometriosis (OR 1.5; 95%CI 1.0–2.3) (Table 3). Σbisphenol concentrations were also associated with increased endometriosis risk (OR 1.5; 95%CI 0.9–2.3), but the association did not reach statistical significance (*p*-value 0.089). When participants were classified by tertiles of BPA and Σbisphenol concentrations, a significantly increased risk was observed for the women in the second versus first tertile [OR 3.7; 95%CI 1.3–10.3 and OR 5.4; 95%CI 1.9–15.6, respectively] (Figure 1), although a significant trend was not observed (*p*-trend > 0.100). As shown in Table 2, there was no statistically significant difference in TBARS concentrations between cases and controls (GM = 3.2 vs. 2.3 μM, *p*-value 0.113); however, multivariate analysis revealed a marginal association between these concentrations and an increased endometriosis risk [OR 1.6, 95%CI 1.0–2.8; *p*-value 0.070], with a significantly increased risk for women in the third versus first tertile of TBARS concentrations (Table 4). In addition, a significant *p*-trend (0.044) was obtained for the adjusted OR across tertiles of TBARS.

TBARS concentrations were positively but non-significantly associated with concentrations of BPA [exp(β) 1.1; 95%CI 1.0–1.3; *p*-value 0.188] and Σbisphenols [exp(β) 1.1; 95%CI 1.0–1.2; *p*-value 0.230] (data not shown in tables). Stratification of TBARS by tertiles showed that these positive associations were only statistically significant for women in the upper tertile (Table 5). The potential confounding effects of other covariates such as educational level, occupational status, or smoking habits were also tested and showed no relevant influence on regression estimates or effect modification on the associations found (data not shown).

## 4. Discussion

This study in women of childbearing age reveals a relationship between inadvertent exposure to bisphenols and the risk of endometriosis, which was greater in the women with higher urinary concentrations of BPA and Σbisphenols. It also contributes novel evidence on the mediating role of lipid peroxidation in this association, which was only statistically significant in the women with the highest urinary TBARS concentration.

The hypothesis that exposure to bisphenols may enhance the risk of endometriosis risk is biologically plausible, as mentioned above. However, there has been little epidemiological research on this association to date, with an increased endometriosis risk reported by some authors [39,40] but no association by others [38,41]. This discrepancy may be attributable to differences in the criteria for selecting participants or in the timing of sample collections. In our study, the presence of endometriosis in cases and its absence in controls was confirmed laparoscopically and histologically, and fasting samples were drawn before surgery. In two of the previous studies [38,39], the absence of endometriotic lesions in controls was not confirmed by laparoscopy, a potential selection bias, and samples were not collected in fasting conditions, a potential measurement bias.

The present findings are in agreement with the study by Rashidi et al. [37], who described an OR of 1.7 (95%CI 1.4–2.2) for the association between urinary BPA concentrations and endometriosis risk after adjustment for age, BMI, parity, and education, although urinary creatinine was not included as a covariate. Simonelli et al. [40] also reported unadjusted associations between exposure and endometriosis, evaluated with the Student’s t-test. This association is also supported by studies of mouse models of endometriosis, which related exposure to bisphenols in adulthood to increased endometriosis lesion growth and atretic oocyte number, disruption of the ovarian steroidogenic pathway, an increase in periglandular fibrosis, and the upregulation of matrix-remodeling enzymes [33,42]. Another in vivo study reported that prenatal exposure to BPA and other bisphenols elicited an endometriosis-like phenotype [43]. Taken together, these in vivo findings suggest that exposure to BPA may be related not only to the development but also to the progression of endometriosis. Higher urinary BPA concentrations were previously observed in women with stage I/II [41] or non-ovarian [39] endometriosis. The number of women with stages I/II or III/IV (n < 25 in each group) was too small for a similar sub-analysis in our study.

Besides a hormonal estrogenic imbalance, a crucial role in the development of endometriosis has been proposed by some authors for oxidative stress, i.e., an imbalance between the production of reactive oxygen/nitrogen species and the ability of biological systems to quickly decode intermediate reagents or repair resulting damage [44]. It has been suggested that oxidative stress may increase the growth and adhesion of endometrial cells in the peritoneal cavity, promoting endometriosis and infertility [29]. In agreement with these studies, we detected higher levels of oxidative lipid damage in cases than in controls. In line with this, it was recently observed that exposure to BPA may be associated with the systemic oxidative damage of macromolecules such as lipids and nucleic acids [45,46] and with disruption of the activity of antioxidant enzymes in specific tissues [23]. It could therefore be hypothesized that oxidative stress may underlie the association between bisphenol exposure and enhanced endometriosis risk. In this regard, a recent in vitro study suggested that the induction of oxidative stress by BPA might be mediated by ER-α in endometrial cells, although it is not fully elucidated whether this action would be in concert with or independent of BPA’s endocrine-disrupting properties [47]. Among oxidative stress biomarkers, TBARS was selected for the present study because it is a good proxy of oxidative status and is a relatively early event, given that the final products possess reactive properties that continue to cause oxidative macromolecule damage [48]. Interestingly, we only observed a significant association between bisphenols and endometriosis in the subset of women in the highest TBARS tertile. Although this has not previously been reported in the setting of endometriosis or other gynecological disorders, it has been suggested that genetic polymorphisms of some oxidative-stress-related genes (e.g., cyclooxygenase 2 (COX2), catalase (CAT), and superoxide dismutase (COX2)) may modify the association between BPA exposure and liver function [49]. It has also been proposed that genetic variants of SOD2 might modify the association between urinary concentrations of phthalates and the risk of asthma, which was increased in patients with the TT SOD2 variant (related to higher oxidative stress levels) [50].

Study limitations include the limited sample size, reducing the statistical power. Nevertheless, statistically significant associations were observed, even after adjustment of the model for numerous potential confounders. Moreover, we cannot rule out that the associations found with single chemicals are a surrogate of exposure to other unmeasured pollutants with similar physicochemical properties or even to mixtures of pollutants that exert a combined effect. In addition, as in previous research on BPA and endometriosis risk, the use of spot urine samples hampers precise characterization of the magnitude of exposure to BPA. On the other hand, unlike some authors, urine samples from all participants (cases and controls) were collected in fasting conditions immediately before surgery, improving the comparability of the magnitude of bisphenol exposure between groups and reducing the potential bias related to intra- and inter-day variabilities in BPA exposure levels. Our findings might also be limited by the collection of urine samples across women’s menstrual cycles, though recent evidence suggests that there is no relationship between menstrual cycle phase and urinary BPA concentrations [51]. Study strengths include the visual and histologic confirmation of the presence of endometriosis in cases and its absence in controls, as already noted, reducing the potential selection bias that may have affected some earlier studies. Furthermore, we provide a more representative overview of the exposure of women to bisphenols by evaluating, to our knowledge for the first time, their exposure to the BPA analogues BPS and BPF. We also offer novel evidence of a mediating role for lipid peroxidation, improving our understanding of the mechanisms underlying the effect of bisphenols on the development of endometriosis.

Taken together, and despite the reduced statistical power, the present results indicate that inadvertent exposure to bisphenols (BPA and substitutes) may increase the risk of endometriosis in women of childbearing age and suggest a potential role for oxidative stress in the endocrine-disruptive effects of BPA in these women. Further studies are warranted to verify our novel findings on the role of oxidative stress as a mediator in the increased risk for endometriosis attributable to bisphenol exposure.

## Figures and Tables

**Table 1:** Characteristics of the study population (n = 128).

		Cases (n = 35)			Controls (n = 89)			*p*-Value
		n		%	n		%	
Age (years) *		38.3	±	9.3	35.8	±	10.4	0.251
Weight (kg) *		68.5	±	13.8	66.4	±	13.6	0.334
Height (m) *		1.6	±	0.1	1.6	±	0.1	0.897
Body mass index (kg/m^2^) *		25.8	±	5.4	25.0	±	4.8	0.671
	Normal weight (BMI < 25)	20		57.1	21		57.3	0.182
	Overweight (BMI 25–30)	6		17.1	26		29.2	
	Obese (BMI > 30)	9		25.7	12		13.5	
Residence								0.156
	Rural	14		40.0	24		27.0	
	Urban /semi-urban	21		60.0	65		73.0	
Parity								0.626
	Nulliparous	16		45.7	37		41.6	
	Primiparous/Multiparous	19		54.3	52		58.4	
Educational level								0.105
	Less than university degree	19		54.3	62		69.7	
	University degree	16		45.7	27		30.3	
Working outside home								0.806
	Yes	20		57.1	53		59.6	
	No	15		42.9	36		40.4	
Current smoker								0.354
	Yes	7		20.0	25		28.1	
	No	28		80.0	64		71.9	
Amount of bleeding								0.366
	Mild	14		40.0	28		31.5	
	Moderate/severe	21		60.0	61		68.5	
Urinary creatinine (ng/mL) *		143.1	±	71.6	115.5	±	61.3	0.052
Endometriosis stage								-
	I	14		40.0	-		-	
	II	9		25.7	-		-	
	III	7		20.0	-		-	
	IV	5		14.3	-		-	
Endometrioma location								
	Deep infiltrating endometriosis	11		31.4	-		-	
	Ovarian/peritoneal endometriosis	24		68.6	-		-	

* Mean ± standard deviation.

**Table 2:** Urinary levels of bisphenols and thiobarbituric acid reactive substances (TBARS) in the study population.

		Total	Cases	Controls	*p*-Value
BPA					
	% ≥ LOD	95.1	100.0	88.5	0.346
	GM (GSD)	3.6 (1.1)	5.5 (1.1)	3.0 (1.2)	
	Min–Max	0.1–47.0	0.8–18.7	0.1–47.0	
	Percentiles 25–75	2.4–8.6	4.0–8.4	1.1–8.8	
BPS					
	% ≥ LOD	14.8	11.4	16.1	0.460
	GM (GSD)	0.1 (1.1)	0.1 (1.1)	0.2 (1.1)	
	Min–Max	0.1–4.2	0.1–1.5	0.1–4.2	
	Percentiles 25–75	0.1–0.1	0.1–0.1	0.1–0.1	
BPF					
	% ≥ LOD	29.5	28.6	29.9	0.532
	GM (GSD)	0.1 (0.1)	0.1 (1.2)	0.1 (1.1)	
	Min–Max	0.1–18.4	0.1–0.9	0.1–18.4	
	Percentiles 25–75	0.1–0.2	0.1–0.2	0.1–0.2	
Σbisphenols					
	GM (GSD)	4.3 (1.1)	5.9 (1.1)	3.8 (1.2)	0.510
	Min–Max	0.2–47.1	0.9–18.9	0.2–47.1	
	Percentiles 25–75	2.8–9.0	4.6–8.5	1.2–10.0	
	% ≥ LOD				
					
TBARS					
	% ≥ LOD	81.5	82.9	80.9	0.113
	GM (GSD)	2.5 (1.1)	3.2 (1.2)	2.3 (1.1)	
	Min–Max	0.1–18.6	0.1–15.0	0.2–18.6	
	Percentiles 25–75	1.3–5.5	1.8–7.0	1.2–5.0	

BPA: bisphenol A; BPS: bisphenol S; BPF: bisphenol F; GM: geometric mean; GSD: geometric standard deviation; LOD: limit of detection.

**Table 3:** Relationship between urinary bisphenols and endometriosis. Logistic regression analyses.

	OR	aOR ^1^	aOR ^2^	aOR ^3^
	(95% CI)	(95% CI)	(95% CI)	(95% CI)
**BPA (ng/mL)**	1.3 (1.0–1.9)	**1.6 (1.0–2.3)**	**1.6 (1.1–2.3)**	**1.5 (1.0–2.3)**
**BPS** *= >LOD*	1.5 (0.5–4.3)	2.6 (0.5–12.4)	2.7 (0.5–13.4)	2.0 (0.4–10.2)
**BPF** *= >LOD*	1.0 (0.4–2.4)	1.3 (0.6–3.4)	1.4 (0.5–3.4)	1.4 (0.5–3.5)
**Σbisphenols (ng/mL)**	***1.5 (1.0–2.3)***	1.5 (1.0–2.3)	1.5 (1.0–2.3)	1.5 (0.9–2.3)

OR: odds ratio; aOR: adjusted OR; BPA: bisphenol A; BPS: bisphenol S; BPF: bisphenol F; LOD: limit of detection; CI: confidence intervals. ^1^ Adjusted for urinary creatinine (mg/dL); ^2^ adjusted for urinary creatinine (mg/dL), age (yr), and body mass index (kg/m^2^); ^3^ adjusted for urinary creatinine (mg/dL), age (yr), body mass index (kg/m^2^), parity (nulliparous/primiparous or multiparous), and residence (rural or suburban/urban). Bold coefficients indicate statistically significant associations (*p* < 0.05)

**Table 4:** Association between urinary TBARS levels and endometriosis.

		Cases (n = 35)	Controls (n = 89)	OR	aOR ^1^	aOR ^2^	aOR ^3^
		n (%)	n (%)	(95% CI)	(95% CI)	(95% CI)	(95% CI)
TBARS				1.4 (0.9–2.1)	1.5 (0.9–2.5)	1.5 (0.9–2.6)	*1.6 (1.0–2.8)*
	*<LOD–1.50*	8 (22.9%)	33 (37.1%)	1.0	1.0	1.0	1.0
	*>1.50–4.23*	11 (31.4%)	30 (33.7%)	1.5 (0.5–4.3)	2.1 (0.6–7.0)	1.7 (0.5–6.1)	2.1 (0.6–7.7)
	*>4.23*	16 (45.7%)	26 (29.2%)	3.5 (0.9–6.8)	**3.7 (1.0–13.5)**	3.6 (1.0–13.2)	**3.8 (1.0–13.9)**

OR: odds ratio; aOR: adjusted OR; TBARS: thiobarbituric acid reactive substances LOD: limit of detection; CI: confidence intervals. ^1^ Adjusted for urinary creatinine (mg/dL); ^2^ adjusted for urinary creatinine (mg/dL), age (yr), and body mass index (kg/m^2^); ^3^ adjusted for urinary creatinine (mg/dL), age (yr), body mass index (kg/m^2^), parity (nulliparous/primiparous or multiparous) and residence (rural or suburban/urban). Bold coefficients indicate statistically significant associations (*p* < 0.05)

**Table 5:** Association between urinary bisphenols and endometriosis by tertiles of urinary TBARS.

	OR	aOR ^1^	aOR ^2^	aOR ^3^
	(95% CI)	(95% CI)	(95% CI)	(95% CI)
TBARS [<1.50 ng/mL]				
BPA (ng/mL)	1.4 (0.6–3.5)	1.1 (0.4–2.9)	0.9 (0.3–2.7)	0.9 (0.3–2.8)
Σbisphenols (ng/mL)	1.3 (0.5–3.6)	0.9 (0.3–2.9)	0.7 (0.2–2.5)	0.7 (0.1–6.9)

TBARS [1.50–4.23 ng/mL]				
BPA (ng/mL)	1.3 (0.7–2.6)	1.3 (0.7–2.6)	1.4 (0.7–3.0)	1.5 (0.7–3.5)
Σbisphenols (ng/mL)	1.2 (0.6–2.4)	1.2 (0.6–2.5)	1.4 (0.6–3.1)	1.4 (0.6–3.5)

**TBARS** [>4.23 ng/mL]				
BPA (ng/mL)	1.9 (1.0–3.5)	**2.0 (1.0–3.8)**	**2.1 (1.0–4.4)**	**2.0 (1.0–4.1)**
Σbisphenols (ng/mL)	**2.0 (1.0–3.9)**	**2.1 (1.0–4.2)**	**2.2 (1.0–4.8)**	**2.2 (1.0–4.6)**

OR: odds ratio; aOR: adjusted OR; TBARS: thiobarbituric acid reactive substances; BPA: bisphenol A; CI: confidence intervals. ^1^ Adjusted for urinary creatinine (mg/dL); ^2^ adjusted for urinary creatinine (mg/dL), age (yr), and body mass index (kg/m^2^); ^3^ adjusted for urinary creatinine (mg/dL), age (yr), body mass index (kg/m^2^), parity (nulliparous/primiparous or multiparous) and residence (rural or suburban/urban). Bold coefficients indicate statistically significant associations (*p* < 0.05)

## Supplementary Materials

The following are available online at https://www.mdpi.com/1660-4601/17/4/1194/s1, Table S1: Urinary levels of bisphenols in controls and cases stratified according to the endometriosis stage.
