# Supplementary material for: Association of Urinary Levels of Bisphenols A, F, and S with Endometriosis Risk: Preliminary Results of the EndEA Study
Source: Int J Environ Res Public Health. 2020 Feb 13;17(4):1194. doi: 10.3390/ijerph17041194 (PMC7068366; doi:10.3390/ijerph17041194)
Supplement: Supplementary file 1 [file ijerph-17-01194-s001.pdf]

Supplementary Table 1. Urinary levels of bisphenols in controls and cases stratified according to the endometriosis stage.

	CONTROLS (n = 89)							CASES (N = 35)																
								Stage I/II (n = 23)							Stage III/IV (n = 12)								
	Mean	SD	Min	Percentiles			Max	Mean	SD	Min	Percentiles			Max	Mean	SD	Min	Percentiles			Max	*p*-value *	*p*-trend #
				P25	P50	P75					P25	P50	P75					P25	P50	P75			
**BPA**	6.4	6.8	0.1	1.1	5.2	8.8	47.0	6.1	4.1	0.8	3.9	5.2	6.7	18.7	7.9	4.5	3.4	4.6	6.5	10.2	18.7	0.299	0.222
**BPS**	0.4	0.8	0.1	0.1	0.1	0.1	4.2	0.2	0.4	0.1	0.1	0.1	0.1	1.5	0.1	0.1	0.1	0.1	0.1	0.1	0.3	0.641	0.485
**BPF**	0.5	2.3	0.1	0.1	0.1	0.2	18.4	0.1	0.2	0.1	0.1	0.1	0.1	0.9	0.3	0.3	0.1	0.1	0.1	0.5	0.9	0.148	0.683
**Σ Bisphenols**	7.3	7.6	0.2	1.2	5.8	10.0	47.1	6.4	4.1	0.9	4.5	5.3	7.7	18.8	8.3	4.4	3.6	5.2	7.5	10.4	18.9	0.391	0.347

* *p*-value from Kruskal-Wallis test. # *p*-trend from Jonckheere-Terpstra test.
